# One-time-pad cipher algorithm based on confusion mapping and DNA storage technology

**DOI:** 10.1371/journal.pone.0245506

**Published:** 2021-01-20

**Authors:** Weiping Peng, Shuang Cui, Cheng Song

**Affiliations:** School of Computer Science and Technology, Henan Polytechnic University, Jiaozuo, Henan, China; University of Engineering & Technology, Taxila, PAKISTAN

## Abstract

In order to solve the problems of low computational security in the encoding mapping and difficulty in practical operation of biological experiments in DNA-based one-time-pad cryptography, we proposed a one-time-pad cipher algorithm based on confusion mapping and DNA storage technology. In our constructed algorithm, the confusion mapping methods such as chaos map, encoding mapping, confusion encoding table and simulating biological operation process are used to increase the key space. Among them, the encoding mapping and the confusion encoding table provide the realization conditions for the transition of data and biological information. By selecting security parameters and confounding parameters, the algorithm realizes a more random dynamic encryption and decryption process than similar algorithms. In addition, the use of DNA storage technologies including DNA synthesis and high-throughput sequencing ensures a viable biological encryption process. Theoretical analysis and simulation experiments show that the algorithm provides both mathematical and biological security, which not only has the difficult advantage of cracking DNA biological experiments, but also provides relatively high computational security.

## Introduction

Since Adleman [1] discovered the computational ability of DNA molecules in 1994, scientists have constructed and assembled DNA molecules in different ways according to biological operations, and implemented logical computational models based on molecular computation using DNA strand replacement, DNA polymerase and nanoparticles [2–4]. In addition, DNA molecules as information carriers begin to be used for DNA storage [5–8] due to it prominent advantages such as large storage capacity, high computational parallelism and low energy consumption. With the rapid development and maturity of DNA synthesis and sequencing technology, DNA storage technology can be realized. It’s a futuristic and epoch-making technology. High-throughput DNA sequencing technology, also known as second-generation sequencing technology, can sequence hundreds of thousands or even millions of DNA molecules at a time. It has been widely used in genomics research since its birth, and is currently an important implementation tool for the construction of DNA storage schemes [9, 10]. The application of DNA molecule in information science not only challenges traditional cryptography which relies on mathematical difficulties for security [11, 12], but also provides conditions for the design of more efficient and reliable DNA cryptography when combined with modern cryptography.

DNA cryptography usually takes DNA as the information carrier and biological technology as the implementation tool to realize the encryption operation method based on DNA technology [13]. The security of encryption scheme based on DNA technology depends on biological difficulties, but there are also some problems such as the unpredictability of experimental results caused by DNA non-specific hybridization and the complexity caused by complicated artificial operation process. In addition, based on some biological characteristics of DNA computation, DNA encryption scheme is implemented through pseudo-DNA computation operations such as encoding mapping, base calculation and confusion encoding table by simulating DNA biological operations [14]. Chaos map is mostly used in image encryption [15–20]. Due to its good pseudo-randomness and unpredictability, sensitivity to initial state and control parameters, the use of chaos map in the pseudo-DNA encryption scheme can improve computational security [14, 21–23]. In a pseudo-DNA cryptography, the biological nature of DNA makes the algorithm more random, but does not provide biosafe because it does not involve biological processes.

In 1917, Gilbert Vernam first proposed one-time-pad. The security of the one-time-pad encryption system depends on the randomness of the key. If the attacker cannot get code book used to encrypt messages, the algorithm is completely confidential. However, it is difficult to generate, store and distribute the key of one-time-pad. Because of DNA its huge storage capacity and high computational parallelism, DNA molecule began to be combined with one-time-pad algorithm to solve the above problems. Therefore, how to solve the complicated and uncontrollable process of biological encryption, and how to better combine pseudo-DNA computing method with one-time-pad are the problems we need to consider. In this paper, a one-time-pad cipher algorithm based on confusion mapping and DNA storage technology is proposed to satisfy both mathematical and biological security.

### Related work

DNA-based one-time-pad cipher algorithms initially used DNA biotechnology to solve key generation, storage and distribution problems. In 2000, Gehani *et al.* [13] designed two one-time-pad DNA encryption schemes using DNA sequence substitution mapping and chip-based DNA microarray technology. This scheme ensures data security from both mathematical and biological aspects, and shows the ultra-high storage density of DNA. But the scheme is difficult to operate in practice. Chen *et al.* [24] also used the massively parallel processing capability of biomolecular computing, in 2003 proposed a cipher design based on the DNA polymerase chain reaction technology. In this scheme, one-time-pad code book is assembled in the form of DNA strand, and modulo 2 addition is performed by primer amplification reaction, which realizes one-time-pad cipher algorithm. However, this algorithm also has some practical difficulties. In 2014, Wang *et al.* [25] also adopted DNA polymerase chain reaction and proposed a one-time-pad encryption algorithm based on DNA cryptography. In the algorithm, the single strands of synthetic DNA are composed of one-time-pad code book and the key distribution problem is solved by polymerase chain reaction. Compared with the afore mentioned cryptographic scheme that only depends on biological experiments, this algorithm proposes a triplet DNA encoding method. The three base combinations correspond to letters, Numbers and symbols, but there are only $C_{64}^{40}$ mapping relationships, so the encoding security is relatively low.

The combination of DNA self-assembly technology and one-time-pad also provides new ideas for DNA cryptography schemes. The one-time-pad encryption algorithms based on DNA tile self-assembly structure are proposed in [26–28]. True key randomness is achieved by the natural process of DNA self-assembly, and each part of the operation is calculated in parallel without human intervention. However, these cryptography schemes only provide biological experimental security, and the security of the scheme will be greatly reduced if an attacker obtains the algorithm operation flow. In 2014, Yang *et al*. [29] proposed a one-time-pad cipher scheme based on the self-assembling structure of DNA, and used the technology of toehold recognition and strand replacement to carry out XOR operation, and finally obtained the fluorescence intensity spectrum of ciphertext. The algorithm has the property of one-time-pad, but there is no corresponding encryption measure for the code book. If the attacker gets the code book and the structure construction method, it is easy to crack the cipher text and get plaintext information. In 2018, Peng *et al*. [30] proposed a one-time-pad scheme that integrates DNA information hiding and three-dimensional DNA self-assembly structure. The self-assembled structure of the scheme generates four random numbers and transmits them in DNA sequence by hiding information. Compared with the scheme proposed in [29], this DNA self-assembly cryptography scheme further ensures the security of self-assembly structure. In addition, DNA self-assembly can be combined with other biotechnologies to form new schemes. In 2017, Li *et al*. [31] proposed a molecular encryption system based on DNA-zyme. The ciphertext and key are encoded into DNA sequence and loaded on a fixed structure. During decrypting, the correct DNA ciphertext strand and fluorescence signal are obtained by adding trigger and DNAzyme structures. DNAzyme-based encryption scheme can better ensure the security of information from the perspective of biotechnology, but due to the limitation of DNAzyme structure, large-scale encryption and decryption cannot be completed.

One-time-pad algorithm can also use DNA biological characteristics to design pseudo-DNA cryptography. In 2014, Wan *et al*. [23] proposed a one-time-pad encryption algorithm based on hyperchaotic DNA computational optimization, which was applied to image encryption. The scheme is based on Logistic hyperchaotic mapping, and DNA base addition and subtraction calculation. Experimental analysis shows that the correlation coefficient of the ciphertext image obtained is close to 0, and the average execution time of encryption and decryption is short. However, the security of the algorithm mainly depends on the key sequence generated according to parameters. If the parameters are leaked and there is no complex encoding mapping and biosecurity to provide support, the algorithm is easy to be cracked. In 2018, Peng *et al*. [14] proposed a one-time-pad algorithm based on multi-base combination mapping coding and DNA computing, and the design types of DNA encoding rules were increased compared with the [23]. In this algorithm, the plaintext is segmented during encryption, and different keys are selected for each segment of plaintext according to different primer pairs. The security of the algorithm is enhanced by simulating the biological experiment process. All the above one-time-pad pseudo-DNA schemes use DNA biological characteristics and experimental process simulation to increase the randomness of the algorithm. If DNA biotechnology can be combined, a more secure and reliable DNA-based one-time-pad cryptography will be formed.

### Our contribution

Most of the DNA-based one-time-pad encryption schemes encoding are relatively simple, and the security of data information transition into DNA information needs to be further enhanced. In addition, the DNA biotechnology involved has some practical operation difficulties or experimental condition limitations. Therefore, the rationality of biological experiments and the high computation security are critical to the design of DNA-based one-time-pad cipher algorithm. Inspiring by the DNA cryptography schemes [9, 30, 32, 33], we proposed a one-time-pad cipher algorithm based on confusion mapping and DNA storage technology. The primary contributions of our constructed algorithm are as below:

1. In the algorithm, the combination of chaos map, encoding mapping, confusion encoding table and the simulation of biological operation process provides a large enough key space, so that the algorithm can effectively resist any form of exhaustive attack. Among them, parameters selection of encoding mapping and confusion coding table ensure the safety of the conversion process of plaintext and DNA sequences, RNA and protein sequences. In addition, simulating the flow of biological information from DNA to proteins makes the algorithm more stochastic.
2. In the encryption and decryption algorithm, security parameters and confounding parameters can be randomly selected in each step. Multiple random steps generate different ciphertext for plaintext, which ensures the dynamic encryption process and provides higher randomness of encryption compared with the above DNA one-time-pad encryption algorithm. Among them, security parameters include two sets of initial Logistic map parameters for generating key, and different key can be generated for every session according to the key generation algorithm.
3. The application of DNA synthesis and high-throughput sequencing technology ensures the biosafety of the algorithm through the physical isolation of plaintext information and biological information. In addition, the DNA library construction method of ciphertext segmentation and reassembly makes it impossible for the attacker to obtain DNA sequence information by sequencing without the adapter sequences. Even if all DNA fragments were sequenced with random primers, DNA ciphertext could not be correctly spliced without index sequences.

### Organization

We will introduce the bases of the cipher algorithm in the next section. In section 3, data and parameters cipher algorithms are formally introduced. In sections 4 and 5, the algorithm is analyzed in detail for performance and security. Finally, we make a summary of the proposed scheme.

## The basics of the algorithm

### Key generation

This paper based on Logistic map and threshold function. Random number generation adopts dual mapping mechanism. The mathematical definition is as follows:

$$\begin{array}{c} x_{i+1}=\mu_{j}x_{i}\left(1-x_{i}\right). \end{array}$$ (1)

$$\begin{array}{c} f\left(x\right)=\{\begin{array}{c} 0,0<x_{i}\leq 0.5\\ 1,0.5<x_{i}<1 \end{array}. \end{array}$$ (2)

In the above formulas, *μ*_*j*_ ∈ [3.569946, 4], *x*_*i*_ ∈ (0, 1). The parameters derivation formula are as follows:

$$\begin{array}{c} \mu_{j}=\mu_{1}+0.001\times \left(j-1\right),\mu_{1}\in \left[3.569946,4\right]. \end{array}$$ (3)

$$\begin{array}{c} g_{j}=\mu_{1}g_{j-1}\left(1-g_{j-1}\right),g_{1}\in \left(0,1\right). \end{array}$$ (4)

By inputting two sets of initial parameters (*μ*_0_, *g*_0_), (*μ*_1_, *g*_1_) in Eqs (3) and (4), the generated parameters are substituted into Eqs (1) and (2) to obtain two sets of random binary sequences *f*(*x*_0_), *f*(*x*_1_), …, *f*(*x*_*n*_), $f\left({x_{0}}^{\prime }\right),f\left({x_{1}}^{\prime }\right),...,f\left({x_{n}}^{\prime }\right)$, where *x*_0_ = *g*_0_, *x*_1_ = *g*_1_. XOR two sets of binary sequences to obtain the key *K*, as shown in Eq (5).

$$\begin{array}{c} K_{i}=f\left(x_{i}\right)⊕f\left({x_{i}}^{\prime }\right),i=0,1,...,n \end{array}$$ (5)

### Encoding mapping

*Encoding rules*: The binary numbers 0, 1 correspond to the bases *A*, *T*/*U*, *C*, *G*. There are 2^*a*^ combinations of *a* binary numbers and 4^*b*^ combinations of *b* bases. If binary numbers and base combinations are one-to-one correspondence, then 2^*a*^ = 4^*b*^. In the case of *a* = 2, there are 4! corresponding relationships between two binary numbers and a single base,as shown in Table 1. And each corresponding relationship is numbered and defined as *key*_*i*_ ∈ [1, 24], *i* = 0, 1, 2.

**Table 1:** Encoding table for *a* = 2.

	**1**	**2**	**3**	**4**	**5**	**6**	**7**	**8**	**9**	**10**	**11**	**12**
A	00	00	00	00	00	00	01	01	01	01	01	01
T/U	01	01	10	10	11	11	00	00	10	10	11	11
C	10	11	01	11	01	10	10	11	00	11	00	10
G	11	10	11	01	10	01	11	10	11	00	10	00
	**13**	**14**	**15**	**16**	**17**	**18**	**19**	**20**	**21**	**22**	**23**	**24**
A	10	10	10	10	10	10	11	11	11	11	11	11
T/U	00	00	01	01	11	11	00	00	01	01	10	10
C	01	11	00	11	00	01	01	10	00	10	00	01
G	11	01	11	00	01	00	10	01	10	00	01	00

In this paper, the encoding rules are used to convert binary sequence to DNA or RNA base sequence respectively.

### Confusion encoding table

In the process of constructing the confusion encoding table and data encryption, a common Arnold scrambling algorithm is used to transform the points (*x*, *y*) to (*x*′, *y*′) in the N × N matrix. The scrambling transformation matrix of Arnold map can be expressed as:

$$\begin{array}{c} {}[\begin{array}{c} x^{\prime }\\ y^{\prime } \end{array}]=[\begin{array}{cc} 1 & m\\ n & mn+1 \end{array}][\begin{array}{c} x\\ y \end{array}]\;\mathrm{mod}\;N. \end{array}$$ (6)

In Eq (6), *x*, *y* ∈ (0, 1, …, *N* − 1), the scrambling parameters *m* and *n* of Arnold map are both positive integers. In Arnold map transformation, the values of the matrix can be uniformly distributed in the scrambled matrix after a certain number of iterations.

Due to the limited encoding mapping relationships, the confusion encoding table is used to enhance the security of the algorithm. The values of *m* and *n* are both 1, and the processes of constructing the confusion encoding table are as follows:

Step1: 16 letters were selected from 20 amino acid letters, and the remaining 4 letters *T*, *V*, *W*, *Y* were randomly added to the amino acid sequence as redundant letters.
Step2: The 16 amino acid letters were selected as rows and columns respectively to construct a 16 × 16 matrix, as shown in Table 2.
Step3: The rows and columns of the 16 × 16 matrix are numbered from 0 to 15, and each value of the matrix is represented by unique coordinate to generate the initial confusion encoding table, as shown in Table 3.
Step4: According to the number of iterations, Arnold map transformation is performed on the initial confusion encoding table. Assuming that Arnold map iteration value is *Pk*, and Arnold map iteration period of 16 × 16 matrix is 12, *Pk* ∈ [0, 12].

**Table 2:** 16 × 16 matrix.

	A	R	N	D	C	Q	E	G	H	I	L	K	M	F	P	S
A	AA	AR	AN	AD	AC	AQ	AE	AG	AH	AI	AL	AK	AM	AF	AP	AS
R	RA	RR	RN	RD	RC	RQ	RE	RG	RH	RI	RL	RK	RM	RF	RP	RS
N	NA	NR	NN	ND	NC	NQ	NE	NG	NH	NI	NL	NK	NM	NF	NP	NS
D	DA	DR	DN	DD	DC	DQ	DE	DG	DH	DI	DL	DK	DM	DF	DP	DS
C	CA	CR	CN	CD	CC	CQ	CE	CG	CH	CI	CL	CK	CM	CF	CP	CS
Q	QA	QR	QN	QD	QC	QQ	QE	QG	QH	QI	QL	QK	QM	QF	QP	QS
E	EA	ER	EN	ED	EC	EQ	EE	EG	EH	EI	EL	EK	EM	EF	EP	ES
G	GA	GR	GN	GD	GC	GQ	GE	GG	GH	GI	GL	GK	GM	GF	GP	GS
H	HA	HR	HN	HD	HC	HQ	HE	HG	HH	HI	HL	HK	HM	HF	HP	HS
I	IA	IR	IN	ID	IC	IQ	IE	IG	IH	II	IL	IK	IM	IF	IP	IS
L	LA	LR	LN	LD	LC	LQ	LE	LG	LH	LI	LL	LK	LM	LF	LP	LS
K	KA	KR	KN	KD	KC	KQ	KE	KG	KH	KI	KL	KK	KM	KF	KP	KS
M	MA	MR	MN	MD	MC	MQ	ME	MG	MH	MI	ML	MK	MM	MF	MP	MS
F	FA	FR	FN	FD	FC	FQ	FE	FG	FH	FI	FL	FK	FM	FF	FP	FS
P	PA	PR	PN	PD	PC	PQ	PE	PG	PH	PI	PL	PK	PM	PF	PP	PS
S	SA	SR	SN	SD	SC	SQ	SE	SG	SH	SI	SL	SK	SM	SF	SP	SS

**Table 3:** Initial confusion encoding table.

	0	1	2	3	4	5	6	7	8	9	10	11	12	13	14	15
0	AA	AR	AN	AD	AC	AQ	AE	AG	AH	AI	AL	AK	AM	AF	AP	AS
1	RA	RR	RN	RD	RC	RQ	RE	RG	RH	RI	RL	RK	RM	RF	RP	RS
2	NA	NR	NN	ND	NC	NQ	NE	NG	NH	NI	NL	NK	NM	NF	NP	NS
3	DA	DR	DN	DD	DC	DQ	DE	DG	DH	DI	DL	DK	DM	DF	DP	DS
4	CA	CR	CN	CD	CC	CQ	CE	CG	CH	CI	CL	CK	CM	CF	CP	CS
5	QA	QR	QN	QD	QC	QQ	QE	QG	QH	QI	QL	QK	QM	QF	QP	QS
6	EA	ER	EN	ED	EC	EQ	EE	EG	EH	EI	EL	EK	EM	EF	EP	ES
7	GA	GR	GN	GD	GC	GQ	GE	GG	GH	GI	GL	GK	GM	GF	GP	GS
8	HA	HR	HN	HD	HC	HQ	HE	HG	HH	HI	HL	HK	HM	HF	HP	HS
9	IA	IR	IN	ID	IC	IQ	IE	IG	IH	II	IL	IK	IM	IF	IP	IS
10	LA	LR	LN	LD	LC	LQ	LE	LG	LH	LI	LL	LK	LM	LF	LP	LS
11	KA	KR	KN	KD	KC	KQ	KE	KG	KH	KI	KL	KK	KM	KF	KP	KS
12	MA	MR	MN	MD	MC	MQ	ME	MG	MH	MI	ML	MK	MM	MF	MP	MS
13	FA	FR	FN	FD	FC	FQ	FE	FG	FH	FI	FL	FK	FM	FF	FP	FS
14	PA	PR	PN	PD	PC	PQ	PE	PG	PH	PI	PL	PK	PM	PF	PP	PS
15	SA	SR	SN	SD	SC	SQ	SE	SG	SH	SI	SL	SK	SM	SF	SP	SS

### DNA structure design for sequencing

**Fig 1:**
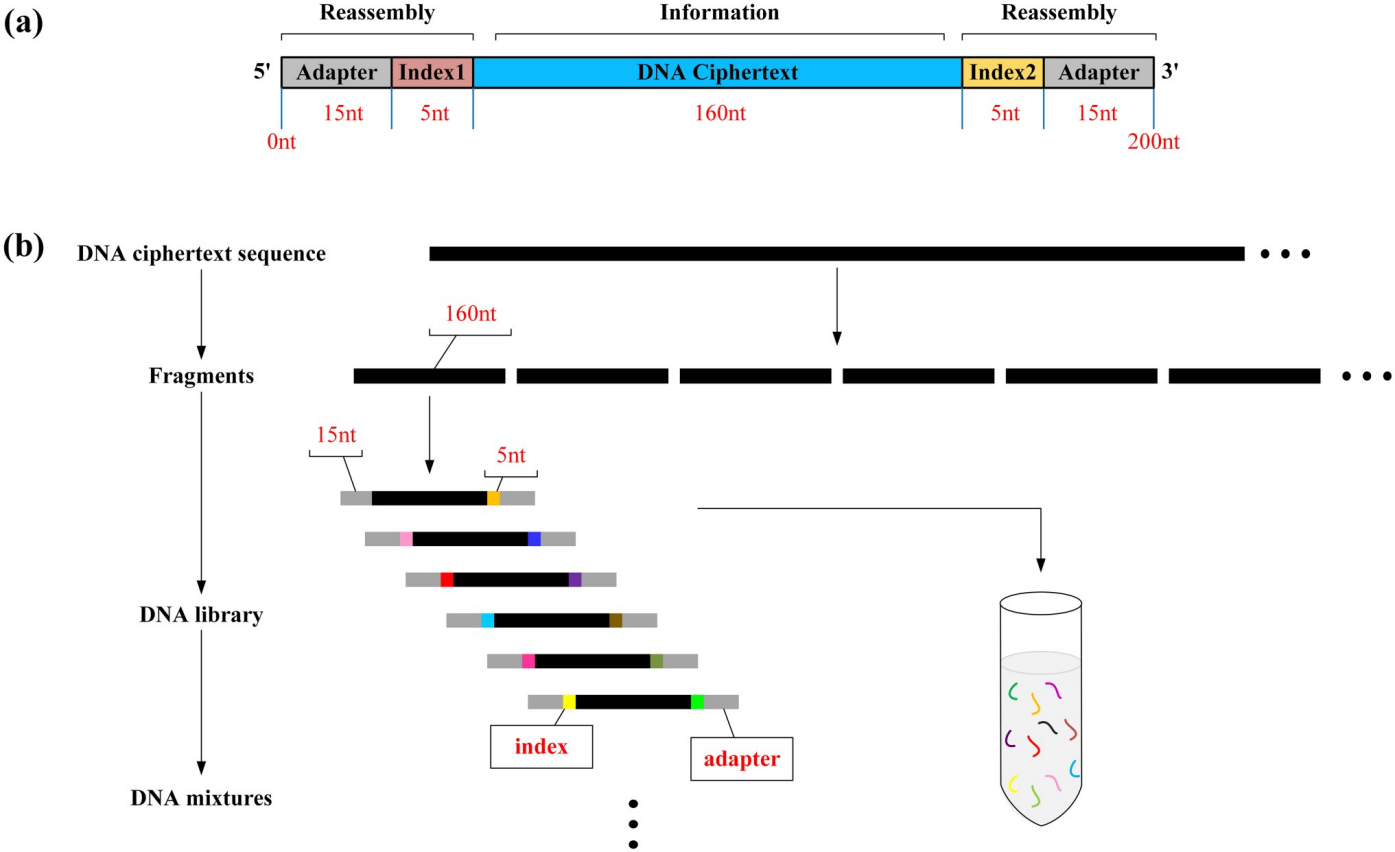
DNA structure design for sequencing. (a) DNA sequencing structure. Each DNA sequencing structure length is 200nt, including 160nt DNA ciphertext information and 20nt reassembly parts at both ends. Reassembly part contains 15nt adapter and 5nt index. (b) Construction of DNA mixtures. The DNA mixtures consists of synthetic DNA sequencing structures.

The DNA structure of high-throughput sequencing contains the reassembly and information regions. The reassembly region is denoted as parameter *r*. Each *r* consists of adapter and index, where the adapter region is used to connect the sequencing primer and the index regions are used to distinguish different samples after sequencing. And the information region is the DNA ciphertext sequence obtained by data encryption. The design of each part of DNA sequencing structure in this scheme is shown in part(a) of the Fig 1.

The sender cuts off the DNA ciphertext sequence obtained after encryption of the plaintext data and adds the adapter and index shared by the receiver at both ends of each DNA ciphertext sequence to generate the DNA library. All the DNA strands are synthesized, mixed, and finally the DNA mixtures are publicly transmitted to the receiver, as shown in part(b) of the Fig 1.

## Proposed algorithm

### Algorithm initialization

Alice randomly selects the parameters used in the encryption process and generates the confusion encoding table. The procedures are as follows:

Step1: Two sets of initial parameters *μ*_0_, *g*_0_ and *μ*_1_, *g*_1_ of Logistic map are randomly selected to obtain the key *K*.
Step2: Encoding mapping parameters *key*_*i*_, *i* = 0, 1, 2 are randomly selected.
Step3: Confusion encoding table parameter *Pk* is randomly selected and the confusion encoding table is generated from the initial confusion encoding table.

### Parameters encryption

#### Security parameters confusion

Alice confuses the security parameters of encoding mapping *key*_0_ and generating key *μ*_0_, *g*_0_, *μ*_1_, *g*_1_.

$$key_{0},\mu_{0},g_{0},\mu_{1},g_{1}\overset{key_{1},key_{2},Pk}{\to }A_{0}\overset{T,V,W,Y}{\to }C_{0}$$

The procedures are as follows:

Step1: Firstly, the security parameters sequence *key*_0_, *μ*_0_, *g*_0_, *μ*_1_, *g*_1_ is converted into binary sequence according to the ASCII table.
Step2: The binary sequence in Setp1 is converted to DNA sequence according to the corresponding mapping relationship *key*_1_ in initialization.
Step3: The DNA sequence generated by Step2 is cut in the middle, the first half is transcribed into mRNA sequence (T→U), and the mRNA sequence is converted into tRNA sequence (A↔U, C↔G). The second half is transcribed into rRNA sequence (T→U).
Step4: tRNA sequence and rRNA sequence are converted into binary sequence according to the mapping relation corresponding to the initialization value *key*_2_.
Step5: 4 bits binary numbers correspond to 1 bit decimal number, tRNA and rRNA binary sequences respectively correspond to decimal sequences *M*_1_, *M*_2_, …, *M*_*i*_ and *N*_1_, *N*_2_, …, *N*_*i*_.
Step6: *M*_1_, *M*_2_, …, *M*_*i*_ and *N*_1_, *N*_2_, …, *N*_*i*_ as the horizontal and vertical coordinate respectively, generating *i* coordinates, and the *i* coordinates (*M*_1_, *N*_1_), (*M*_2_, *N*_2_), …, (*M*_*i*_, *N*_*i*_) generate amino acid sequence *A*_0_ according to the confusion encoding table.
Step7: Redundant amino acid letters *T*, *V*, *W*, *Y* are randomly added to amino acid sequence *A*_0_ to generate amino acid ciphertext sequence *C*_0_.

**Fig 2:**
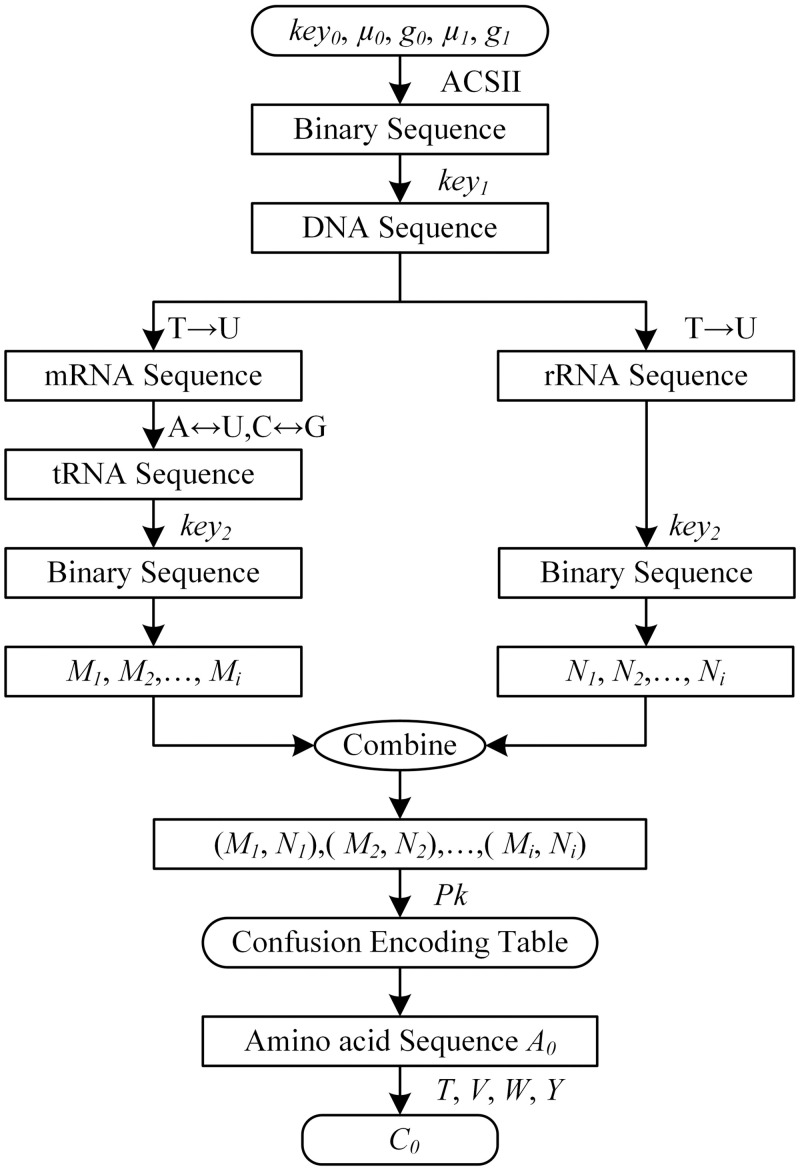
Security parameters confusion.

The above security parameters confusion process simulates the flow of genetic information from DNA to protein through RNA. It is that the process of DNA transcription to mRNA and mRNA translation to protein with specific amino acid sequence. The encryption processes are shown in Fig 2.

#### Confounding parameters encryption

Alice encrypts the confounding parameters *key*_1_, *key*_2_ and *Pk*, which are used for the security parameter confusion.

$$key_{1},key_{2},Pk\overset{Pk=0}{\to }A_{1}\overset{T,V,W,Y}{\to }C_{1}$$

The procedures are as follows:

Step8: According to ASCII code, the confounding parameters sequence *key*_1_,*key*_2_, *Pk* is transformed into binary sequence.
Step9: 4 bits binary numbers correspond to 1 bit decimal number, the first and second halves of the decimal sequence generated by the binary sequence are *M*_1_, *M*_2_, …, *M*_*j*_ and *N*_1_, *N*_2_, …, *N*_*j*_.
Step10: The decimal sequences *M*_1_, *M*_2_,…,*M*_*j*_ and *N*_1_, *N*_2_, …, *N*_*j*_ as the horizontal and vertical coordinate respectively, generating *j* coordinates, and the *j* coordinates (*M*_1_, *N*_1_), (*M*_2_, *N*_2_), …, (*M*_*j*_, *N*_*j*_) generate amino acid sequence *A*_1_ according to the confusion encoding table.
Step11: Redundant amino acid letters *T*, *V*, *W*, *Y* are randomly added to amino acid sequence *A*_1_ to generate amino acid ciphertext sequence *C*_1_.

**Fig 3:**
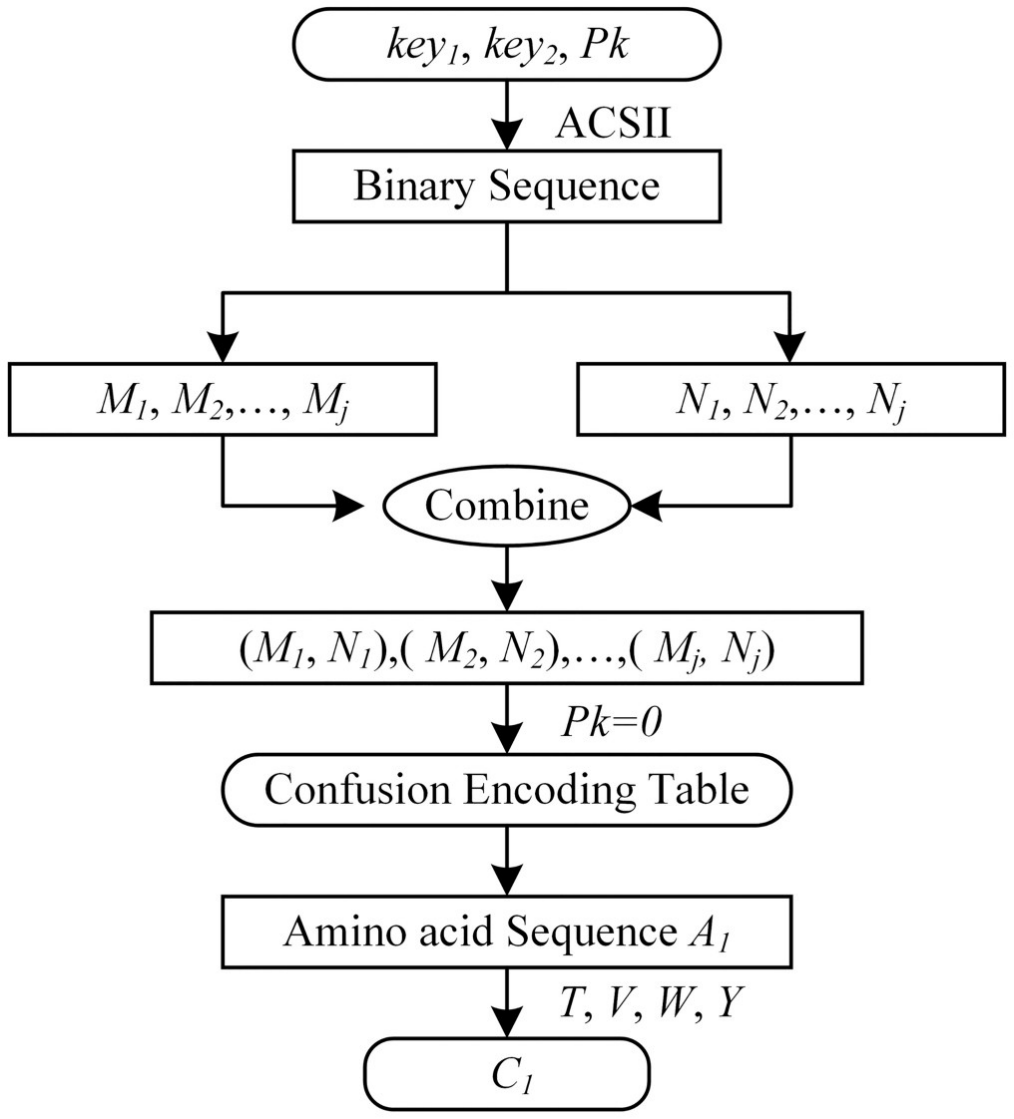
Confounding parameters encryption.

The confounding parameters encryption processes are shown in Fig 3.

#### Parameters ciphertext generation

Alice transmits the amino acid parameters ciphertext, including the amino acid ciphertext *C*_0_ generated by the security parameters confusion and the amino acid ciphertext *C*_1_ generated by the confounding parameters encryption, to Bob through the public channel.

### Data encryption

Alice encrypts the plaintext with the key *K*, encoding mapping parameter *key*_0_, the adapter and index sequence of DNA sequencing structure. And the resulting DNA mixtures are transmitted to Bob through the public channel.

$$P\overset{key_{0},\mu_{0},g_{0},\mu_{1},g_{1}}{\to }DNA\;mixtures$$

The procedures are as follows:

Step1: According to the ASCII code, converting plaintext sequence *P* into binary plaintext sequence *P*_*b*_.
Step2: XOR *P*_*b*_ with the *K* to obtain binary ciphertext sequence *C*_*b*_.
Step3: *C*_*b*_ is divided into *x* groups. Each group length is 2^18^ bits, and converted into a 512 × 512 binary matrix. These binary matrices are represented as *C*_*b*1_, *C*_*b*2_, …, *C*_*bx*_.
Step4: *C*_*b*1_, *C*_*b*2_, …, *C*_*bx*_ are respectively converted to *C*_*B*1_, *C*_*B*2_, …, *C*_*Bx*_ by Arnold map.
Step5: *C*_*B*1_, *C*_*B*2_, …, *C*_*Bx*_ are transformed into the binary sequences in turn, and the final binary ciphertext sequence *C*_*B*_ is obtained.
Step6: Convert *C*_*B*_ to the DNA ciphertext sequence *C*_*DNA*_ according to *key*_0_.
Step7: Divide *C*_*DNA*_ into *n* sections, each segment length is 160nt, and obtain *C*_*DNA*1_, *C*_*DNA*2_, …, *C*_*DNAn*_.
Step8: Add reassembly parameter *r* shared by the receiver to both ends of each DNA ciphertext sequence, including adapter and index sequence, to obtain *n* segments DNA sequence{*r*_1_, *C*_*DNA*1_, *r*_2_}, {*r*_3_, *C*_*DNA*2_, *r*_4_}, …, {*r*_2*n*−1_, *C*_*DNAn*_, *r*_2*n*_}.
Step9: The synthetic DNA sequences {*r*_1_, *C*_*DNA*1_, *r*_2_}, {*r*_3_, *C*_*DNA*2_, *r*_4_},…, {*r*_2*n*−1_, *C*_*DNAn*_, *r*_2*n*_} are mixed and transmitted to receiver Bob through the public channel.

The pseudo code of the data encryption processes are as follows:

**Algorithm 1** Data encryption

**Require:** Plaintext *P*; Binary key *K*; Encoding mapping parameter *key*_0_; Reassembly parameter *r*

**Ensure:** DNA mixtures

1: [ASCII values]: = convert plaintext *P* into ASCII values;

2: [*P*_*b*_]: = transform ASCII values to binary plaintext *P*_*b*_;

3: [*C*_*b*_]: = XOR *P*_*b*_ with *K* to obtain binary ciphertext sequence *C*_*b*_;

4: [*C*_*b*1_, *C*_*b*2_, …*C*_*bx*_]: = *C*_*b*_ is divided into *x* groups binary matrices *C*_*b*1_, *C*_*b*2_, …*C*_*bx*_;

5: [*C*_*B*1_, *C*_*B*2_, …*C*_*Bx*_]: = *C*_*b*1_, *C*_*b*2_, …*C*_*bx*_ are converted to *C*_*B*1_, *C*_*B*2_, …*C*_*Bx*_ by Arnold map;

6: [*C*_*B*_]: = *C*_*B*1_, *C*_*B*2_, …*C*_*Bx*_ are transformed into the final binary ciphertext sequence *C*_*B*_;

7: [*C*_*DNA*_]: = convert *C*_*B*_ into DNA ciphertext *C*_*DNA*_ according to *key*_0_;

8: [*C*_*DNA*1_, *C*_*DNA*2_, …, *C*_*DNAn*_]: = *C*_*DNA*_ is divided into *n* segments to obtain *C*_*DNA*1_,*C*_*DNA*2_,…,*C*_*DNAn*_;

9: [DNA library]: = The reassembly parameter *r* is added for each ciphertext sequence in turn to obtain DNA library {*r*_1_, *C*_*DNA*1_, *r*_2_}, {*r*_3_, *C*_*DNA*2_, *r*_4_},…, {*r*_2*n*−1_, *C*_*DNAn*_, *r*_2*n*_};

10: [DNA mixtures]: = DNA library are synthesized, mixed and transmitted to receiver Bob through the public channel.

### Parameters decryption

#### Confounding parameters decryption

Bob first decrypts the confounding parameters *key*_1_, *key*_2_ and *Pk*.

$$C_{1}\overset{T,V,W,Y}{\to }A_{1}\overset{Pk=0}{\to }key_{1},key_{2},Pk$$

The procedures are as follows:

Step1: Redundant amino acid letters *T*, *V*, *W*, *Y* are removed from amino acid ciphertext sequence *C*_1_ to obtain amino acid sequence *A*_1_.
Step2: The horizontal and vertical coordinate sequences *M*_1_, *M*_2_, …, *M*_*j*_ and *N*_1_, *N*_2_, …, *N*_*j*_ are restored from *A*_1_ according to the initial confusion coding table.
Step3: The decimal sequence *M*_1_, *M*_2_, …, *M*_*j*_, *N*_1_, *N*_2_, …, *N*_*j*_ is restored to the binary sequence.
Step4: According to the ASCII codes, parameters *key*_1_, *key*_2_ and *Pk* are restored.

**Fig 4:**
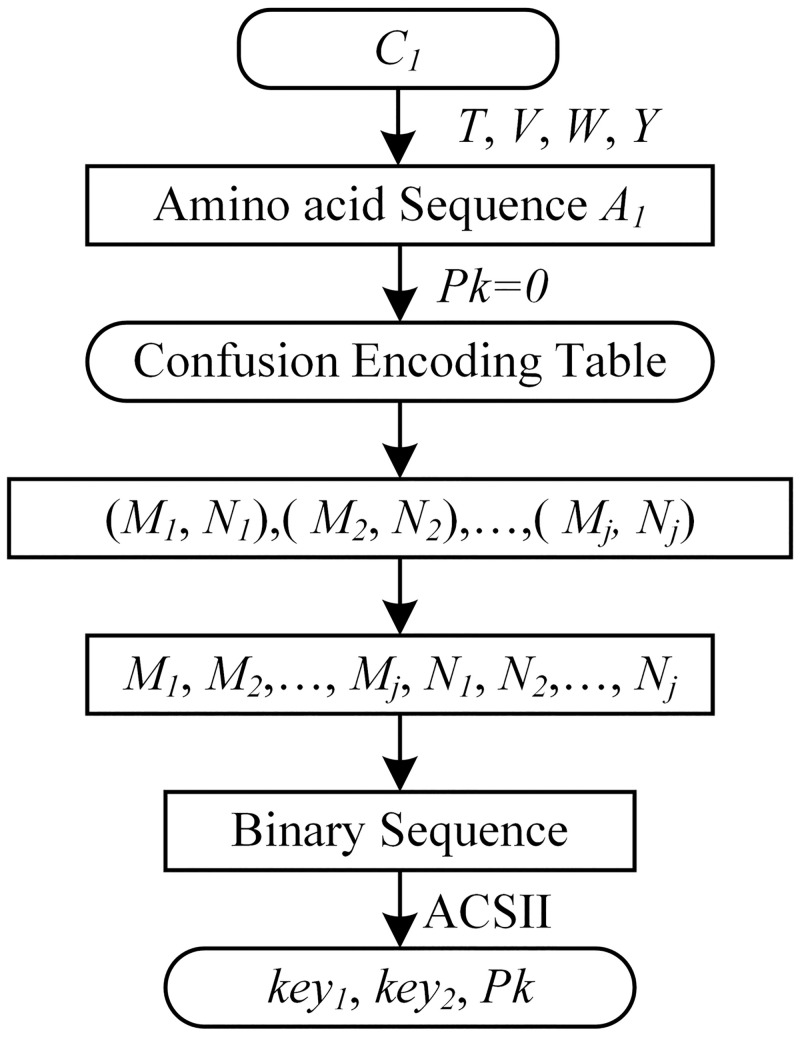
Confounding parameters decryption.

The confounding parameters decryption processes are shown in Fig 4.

#### Security parameters decryption

Bob decrypts the security parameters *key*_0_, *μ*_0_, *g*_0_, *μ*_1_, *g*_1_.
  $$C_{0}\overset{T,V,W,Y}{\to }A_{0}\overset{key_{1},key_{2},Pk}{\to }key_{0},\mu_{0},g_{0},\mu_{1},g_{1}$$
Step5: Redundant amino acid letters *T*, *V*, *W*, *Y* are removed from amino acid ciphertext sequence *C*_0_ to obtain amino acid sequence *A*_0_.
Step6: The horizontal and vertical coordinate sequences *M*_1_, *M*_2_, …, *M*_*i*_ and *N*_1_, *N*_2_, …, *N*_*i*_ are restored from *A*_0_ according to the confusion coding table.
Step7: The decimal sequence *M*_1_, *M*_2_, …, *M*_*i*_ and *N*_1_, *N*_2_, …, *N*_*i*_ can be reduced to obtain tRNA and rRNA binary sequences.
Step8: Transform tRNA and rRNA binary sequences into tRNA and rRNA sequences respectively according to the mapping relationship corresponding to *key*_2_.
Step9: tRNA sequence is converted into mRNA sequence (A↔U,C↔G), mRNA sequence and rRNA sequence are merged and then reverse transcribed into DNA sequence (U→T).
Step10: Convert DNA sequence into binary sequence according to the mapping relation corresponding to *key*_1_.
Step11: According to ASCII code, the value of *key*_0_ and *μ*_0_, *g*_0_, *μ*_1_, *g*_1_ are restored.

**Fig 5:**
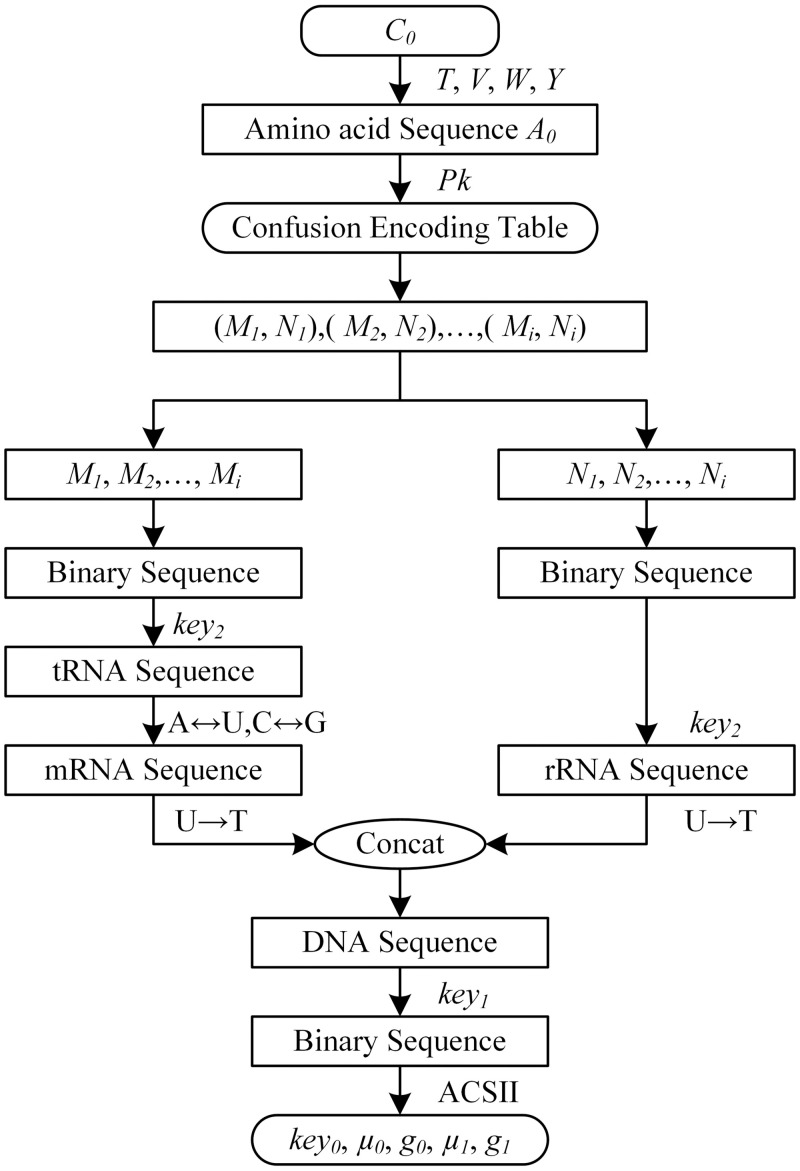
Security parameters decryption.

The security parameters decryption processes are shown in Fig 5.

### Data decryption

After sequenced the DNA mixtures, splicing to obtain the DNA ciphertext sequence *C*_*DNA*_ according to the index sequence. And then decrypted the plaintext data according to the security parameters *key*_0_, *μ*_0_, *g*_0_, *μ*_1_, *g*_1_.

$$DNA\;mixtures\overset{key_{0},\mu_{0},g_{0},\mu_{1},g_{1}}{\to }P$$

The procedures are as follows:

Step1: According to two sets of different initial parameters *μ*_0_, *g*_0_ and *μ*_1_, *g*_1_ to obtain the binary key sequence *K*.
Step2: High-throughput sequencing technology is used to obtain the *n* segments indexed DNA sequences{*i*_1_, *C*_*DNA*1_, *i*_2_}, {*i*_3_, *C*_*DNA*2_, *i*_4_}, …, {*i*_2*n*−1_, *C*_*DNAn*_, *i*_2*n*_}.
Step3: DNA ciphertext sequence *C*_*DNA*_ is correctly spliced through known index sequences.
Step4: Convert *C*_*DNA*_ into the final binary ciphertext sequence *C*_*B*_ according to *key*_0_.
Step5: *C*_*B*_ is divided into *x* groups. Each group length is 2^18^ bits, and converted into a 512 × 512 binary matrix. These binary matrices are represented as *C*_*B*1_, *C*_*B*2_, …*C*_*Bx*_.
Step6: *C*_*B*1_, *C*_*B*2_, …*C*_*Bx*_ are respectively converted to *C*_*b*1_, *C*_*b*2_, …*C*_*bx*_ by Arnold map decrypting.
Step7: *C*_*b*1_, *C*_*b*2_, …*C*_*bx*_ are transformed into the binary sequences in turn, and the binary ciphertext sequence *C*_*b*_ is obtained.
Step8: The binary plaintext sequence *P*_*b*_ is obtained by XOR *C*_*b*_ with *K*.
Step9: According to the ASCII code, converting *P*_*b*_ to plaintext sequence *P*.

**Fig 6:**
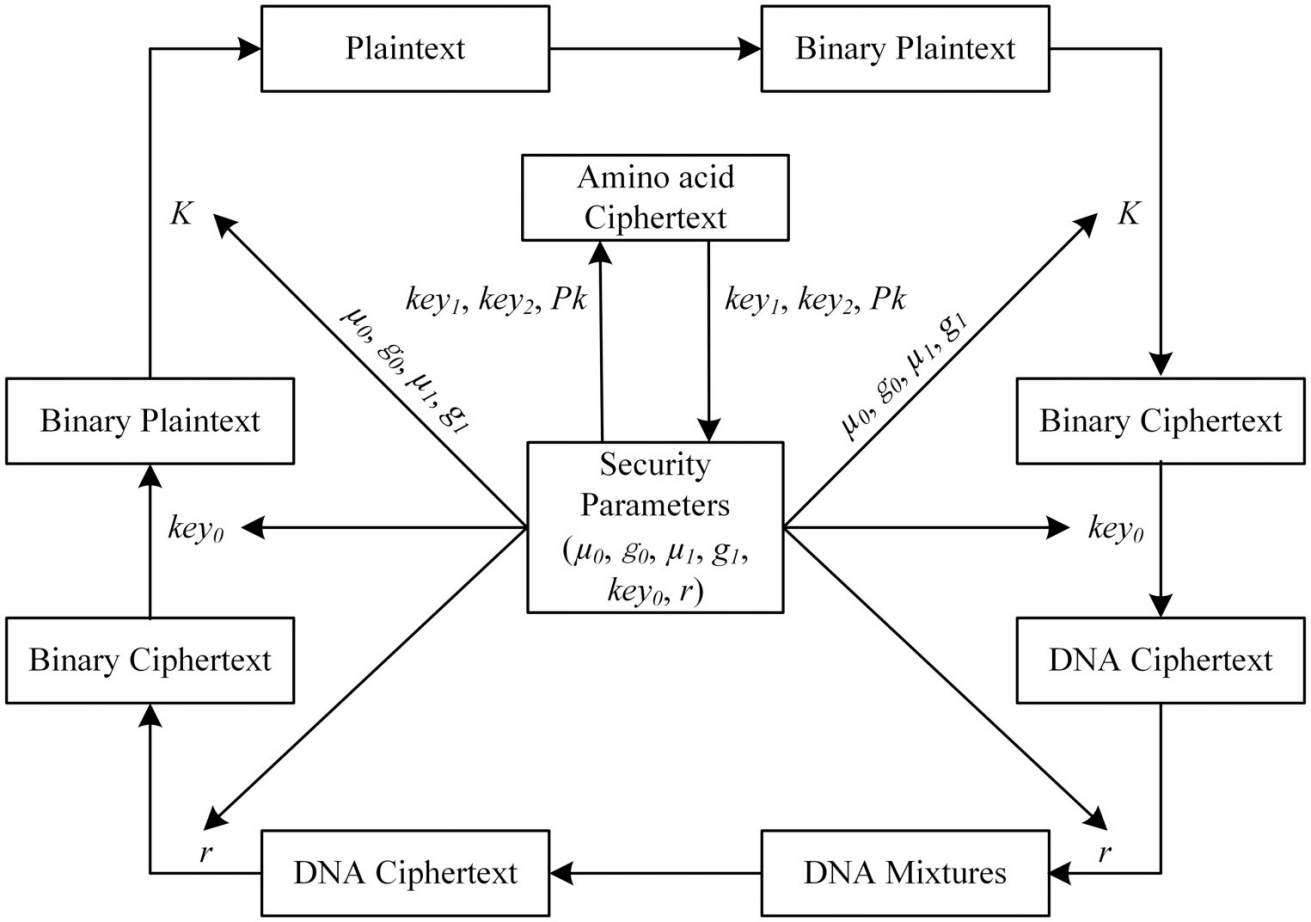
Flow chart of encryption and decryption algorithm. The algorithm includes data and parameters cipher. The security parameters used in data cipher algorithm include *key*_0_, *μ*_0_, *g*_0_, *μ*_1_, *g*_1_, *r*, and the confounding parameters used in parameter cipher algorithm include *key*_1_, *key*_2_, *Pk*.

The pseudo code for the data decryption processes are shown in Algorithm 2. And the processes of cipher algorithm are shown in Fig 6.

**Algorithm 2** Data Decryption

**Require:** DNA mixtures; Logistic map initial parameters *μ*_0_, *g*_0_, *μ*_1_, *g*_1_; Encoding mapping parameter *key*_0_

**Ensure:** Plaintext *P*

1: [chaos sequences]: = generating two chaotic sequences with substituting two initial values *μ*_0_, *g*_0_, *μ*_1_, *g*_1_ into the Logistic formula;

2: [*K*]: = XOR two chaotic sequences to form the key sequence *K*;

3: [Indexed DNA sequences]: = high-throughput sequencing is used to sequence DNA mixtures and obtain indexed DNA sequences {*i*_1_, *C*_*DNA*1_, *i*_2_},{*i*_3_, *C*_*DNA*2_, *i*_4_}, …, {*i*_2*n*−1_, *C*_*DNAn*_, *i*_2*n*_};

4: [*C*_*DNA*_]: = The DNA ciphertext sequence *C*_*DNA*_ is correctly spliced by known index sequences;

5: [*C*_*B*_]: = convert *C*_*DNA*_ into binary ciphertext sequence *C*_*B*_ according to *key*_0_;

6: [*C*_*B*1_, *C*_*B*2_, …*C*_*Bx*_]: = *C*_*B*_ is transformed into *x* groups binary matrices *C*_*B*1_, *C*_*B*2_, …*C*_*Bx*_;

7: [*C*_*b*1_, *C*_*b*2_, …*C*_*bx*_]: = *C*_*B*1_, *C*_*B*2_, …*C*_*Bx*_ are respectively converted to *C*_*b*1_, *C*_*b*2_, …*C*_*bx*_ by Arnold map decrypting;

8: [*C*_*b*_]: = *C*_*b*1_, *C*_*b*2_, …*C*_*bx*_ are transformed into the binary ciphertext sequence *C*_*b*_;

9: [*P*_*b*_]: = The binary plaintext sequence *P*_*b*_ is obtained by XOR *C*_*b*_ with *K*;

10: [ACSII values]: = convert *P*_*b*_ into ACSII values;

11: [*P*]: = transform ACSII values to plaintext *P*.

## Performance analysis

### Parameters frequency analysis

Detailed example: The security parameters selected randomly include the encoding mapping parameter *key*_0_ = 12 and two different sets of Logistic map initial parameters (*μ*_0_, *g*_0_) = (3.78, 0.51), (*μ*_1_, *g*_1_) = (3.92, 0.44). The confounding parameters selected randomly include the encoding mapping parameters *key*_1_ = 4, *key*_2_ = 7 and confusion encoding table parameter *Pk* = 1. The specific encryption processes of parameters are as follows:

Step1: According to the ASCII code, first of all, the security parameters sequence 12,3.78,0.51,3.92,0.44 is converted into binary sequence.
  00110001 00110010 00101100 00110011 00101110 00110111 00111000 00101100
  00110000 00101110 00110101 00110001 00101100 00110011 00101110 00111001
  00110010 00101100 00110000 00101110 00110100 00110100.
Step2: Convert the binary sequence in Setp1 into DNA sequence according to the mapping relationship corresponding to *key*_1_ = 4.
  ACAG ACAT ATCA ACAC ATCT ACGC ACTA ATCA ACAA ATCT ACGG
  ACAG ATCA ACAC ATCT ACTG ACAT ATCA ACAA ATCT ACGA ACGA.
Step3: Divide the DNA sequences generated by Step2 into the middle. The first half can be transcribed to mRNA sequences (T→U), and then the mRNA sequences can be converted to tRNA sequences (A↔U,C↔G).
  UGUC UGUA UAGU UGUG UAGA UGCG UGAU UAGU UGUU UAGA UGCC
  and the second half is transcribed into rRNA sequences
  ACAG AUCA ACAC AUCU ACUG ACAU AUCA ACAA AUCU ACGA ACGA.
Step4: tRNA sequence and rRNA sequence are converted to binary sequence respectively according to the mapping relationship corresponding to *key*_2_ = 7.
  00110010 00110001 00011100 00110011 00011101 00111011 00110100 00011100
  00110000 00011101 00111010 and 01100111 01001001 01100110 01001000 01100011
  01100100 01001001 01100101 01001000 01101101 01101101.
Step5: 4 bits binary numbers correspond to 1 bit decimal number, tRNA and rRNA binary sequences respectively correspond to decimal sequences.
  3 2 3 1 1 12 3 3 1 13 3 11 3 4 1 12 3 0 1 13 3 10 and 6 7 4 9 6 6 4 8 6 3 6 4 4 9 6 5 4
  8 6 13 6 13.
Step6: The two sets of decimal sequences as the horizontal and vertical coordinate respectively, generating 22 coordinates. And the coordinates generate amino acid sequence *A*_0_ according to the confusion encoding table.
  AD FQ NR IH MQ NL NR PQ MQ GE AD NI NR SQ MQ DI NR HH MQ FA AD
  GD.
Step7: Redundant amino acid letters *T*, *V*, *W*, *Y* are randomly added to amino acid sequence to generate amino acid ciphertext sequence *C*_0_.
  ADFVQNRIHYVMQWTNLNRPQMQGEVADNINRSYQMWQDIVNRTHHMQFAA
  DWGTD.
Step8: According to ASCII code, the confounding parameters sequence 4,7,1 is transformed into binary sequence.
  00110100 00101100 00110111 00101100 00110001.
Step9: 4 bits binary numbers correspond to 1 bit decimal number, the first and second halves of the decimal sequence generated by the binary sequence.
  3 4 2 12 3 and 7 2 12 3 1.
Step10: The two sets of decimal sequences are abscissa and ordinate respectively, generating 5 coordinates. And the coordinates generate amino acid sequence *A*_1_ according to the initial confusion encoding table.
  DG CN NM MD DR.
Step11: Redundant amino acid letters *T*, *V*, *W*, *Y* are randomly added to amino acid sequence *A*_1_ to generate amino acid ciphertext sequence *C*_1_.
  DTGCNNMYMDWDR.
Step12: Final parameters ciphertext *C*_0_ and *C*_1_.
  ADFVQNRIHYVMQWTNLNRPQMQGEVADNINRSYQMWQDIVNRTHHMQFAA
  DWGTD and DTGCNNMYMDWDR.

**Fig 7:**
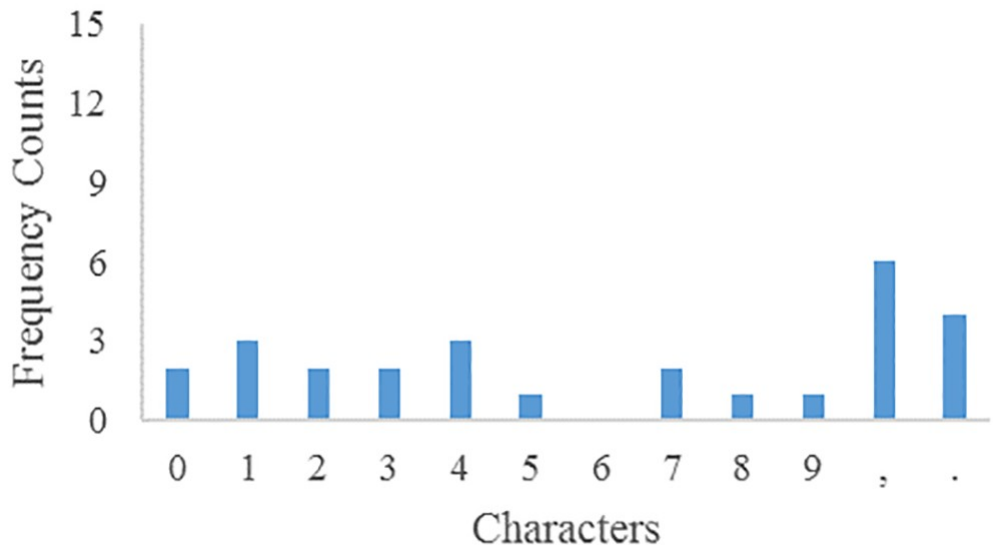
Parameters plaintext characters frequency.

**Fig 8:**
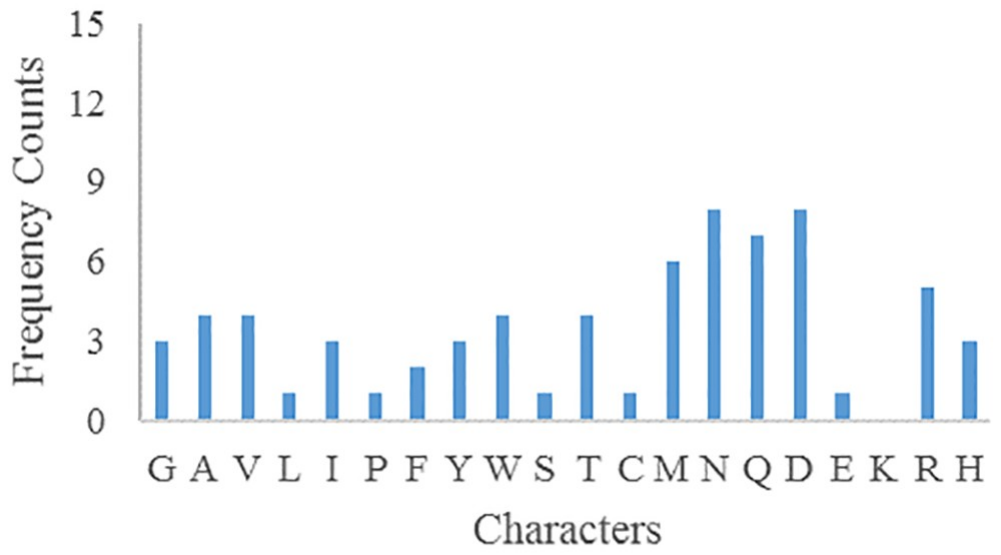
Parameters ciphertext characters frequency.

The frequency counts of parameters plaintext and ciphertext characters are shown in Figs 7 and 8 respectively. After encryption, the frequency of parameters ciphertext is very different from that of plaintext, so there is no correlation.

**Fig 9:**
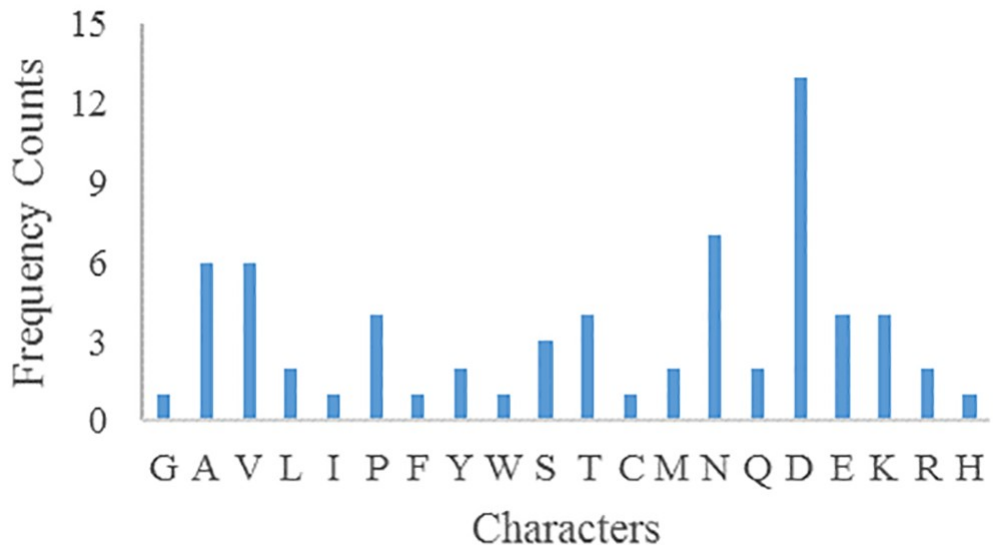
Parameters ciphertext characters frequency for *key*_1_ = 5.

Assuming that *key*_1_ = 5, and other confounding parameters remain unchanged, the frequency of ciphertext characters is shown in Fig 9.

**Fig 10:**
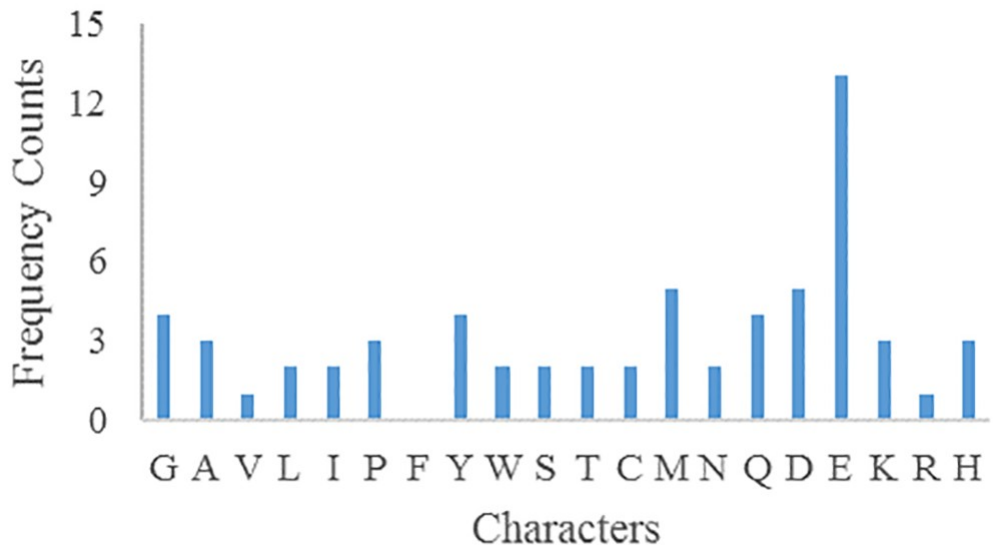
Parameters ciphertext characters frequency for *key*_2_ = 6.

Assuming that *key*_2_ = 6, and other confounding parameters remain unchanged, the frequency of ciphertext characters is shown in Fig 10.

**Fig 11:**
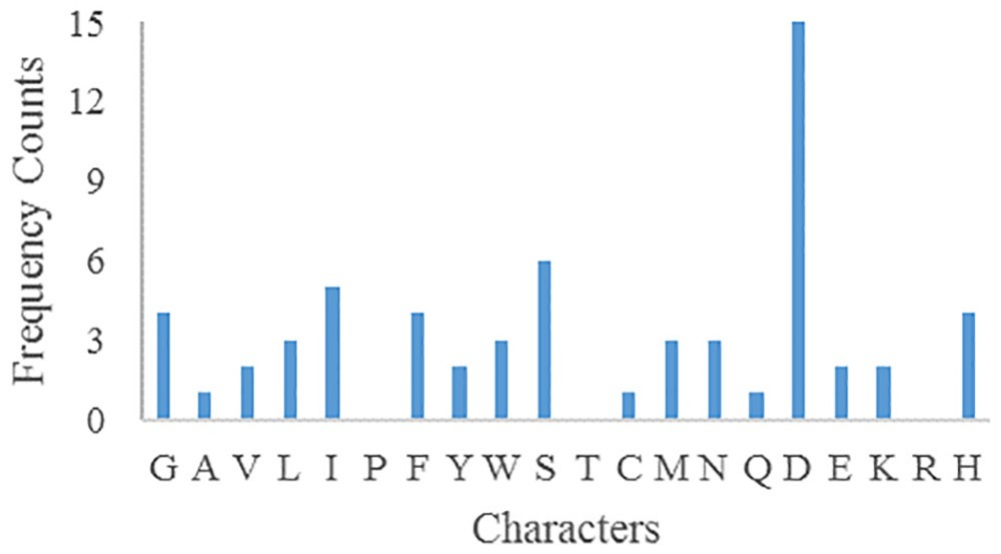
Parameters ciphertext characters frequency for *Pk* = 2.

Assuming that *Pk* = 2, and other confounding parameters remain unchanged, the frequency of ciphertext characters is shown in Fig 11.

The above three confounding parameters *key*_1_, *key*_2_, *Pk* were slightly changed, and the parameters ciphertext characters frequencies were greatly different from that before, and there was no correlation, which could resist statistical analysis.

### Data frequency analysis

**Fig 12:**
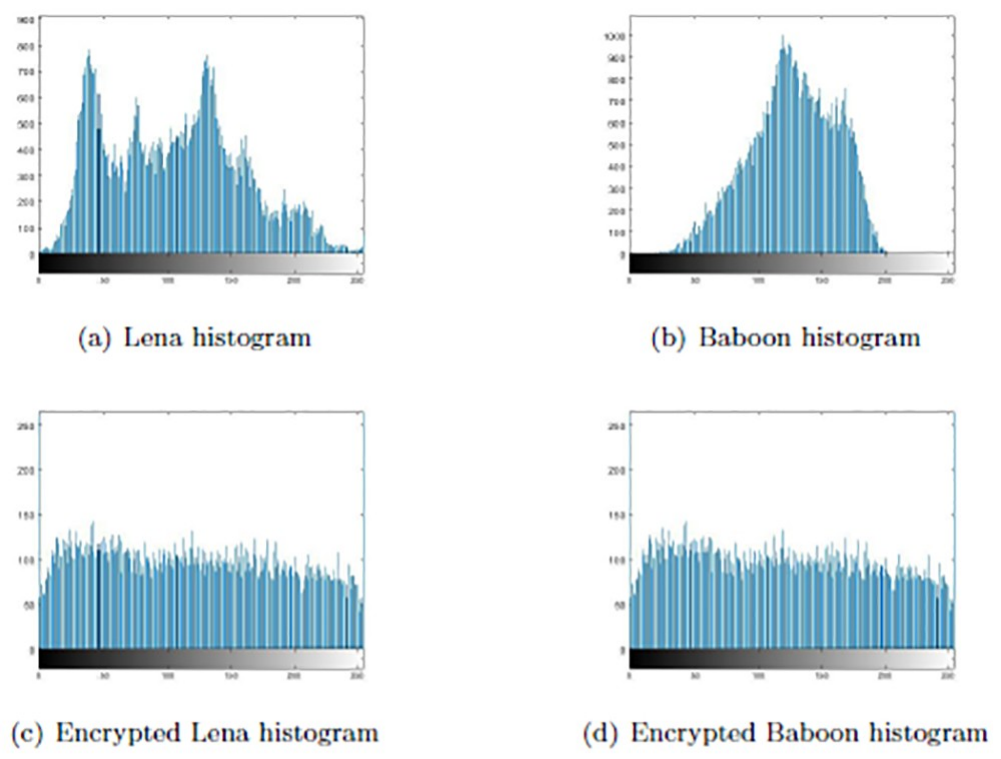
Frequency analysis of original and encrypted image. (a) and (b) are original image pixel frequency histograms. (c) and (d) are encrypted image pixel frequency histograms.

In the data encryption, binary plaintext converting to binary ciphertext with one-time-pad key *K* and Arnold map encryption. In order to test the statistical relationship between binary plaintext and ciphertext, gray image was selected as the source data for encryption simulation experiment. The computer was configured as 2.50GHz processor, 8GB memory, Windows 7 operating system and the simulation software was Matlab R2018b. Two sets of Logistic map initial parameters (*μ*_0_, *g*_0_) = (3.78, 0.51) and (*μ*_1_, *g*_1_) = (3.92, 0.44) were selected, and the *K* was obtained by substituting into the key generation algorithm. For example, we select the gray images Lena and Baboon with the size of 160 × 160 pixels. The distribution of images pixel frequency is analyzed statistically before and after encryption, as shown in Fig 12. By comparing the histograms before and after encryption, it can be seen that the features of the original image after encryption are effectively concealed and there was no statistical similarity between the histogram of original and encrypted pixel frequency.

We calculate variances of histograms to evaluate uniformity of ciphered images. The lower value of variance between two different ciphertext images indicates the higher uniformity of encrypted image. We employ the variance formula in reference [34], as shown in Eq (7).

$$\begin{array}{c} \mathrm{var}\left(Z\right)=\frac{1}{n^{2}}\sum_{i=1}^{n}\sum_{j=1}^{n}\frac{1}{2}{\left(z_{i}-z_{j}\right)}^{2}. \end{array}$$ (7)

For quantity analyses of each Logistic map initial parameters, we calculated the images variance encrypted with different parameters on the same plaintext image. Based on the logistic map initial parameters of the above the generated key *K*, only one parameter value is changed at a time to compare the variance value of ciphertext images. The closer of the two variance values are, the better uniformity of the encrypted image is when the parameters changes. The variance of ciphered images compared among all parameters is shown in Table 4. The variance values of ciphertext images are about 5000, and encrypting the same plaintext image with different parameters results in a small difference in the variance values of the encrypted image. Therefore, the data encryption algorithm is efficient.

**Table 4:** Variance analysis for the uniformity of encrypted image.

Ciphered image	*μ*_0_ = 3.77	*g*_0_ = 0.405	*μ*_1_ = 3.85	*g*_1_ = 0.73
Lena	4585.85	4541.05	5328.00	5201.00
Baboon	4585.60	4540.85	5327.00	5200.50

The information entropy can reflect the pixel distribution of the gray image. The formula for calculating the entropy of encrypted image is as follows:

$$\begin{array}{c} H\left(x\right)=-\sum_{i=0}^{255}P(x_{i})\log_{2}P\left(x_{i}\right). \end{array}$$ (8)

When the entropy value of the encrypted image is close to 8, the distribution of gray histogram becomes more uniform. Different initial parameters are used to encrypt the images for 5 times and the average entropy of ciphered images is shown in Table 5. The encrypted image entropy value *H*(*x*) calculated according to Eq (8) is close to 8. Therefore, data encryption can effectively resist statistical attacks.

**Table 5:** Information entropy of encrypted image.

Image data	size	Encrypted image entropy
Lena	160 × 160	7.9376
Baboon	160 × 160	7.9357

Lena gray images of different sizes are selected to test the encryption time. Different initial values are used to encrypt for 5 times and the average encryption time is compared with the recently image encryption algorithms [18–20], as shown in Table 6. The comparison results indicate that the encryption time of our algorithm is reasonable and effective.

**Table 6:** Encryption time comparison(s).

Image size	Ref. [18]	Ref. [19]	Ref. [20]	Our algorithm
256 × 256	0.16	0.11794	0.0779	0.0816244
512 × 512	0.62	0.27444	0.3261	0.3415876

### Algorithm time complexity analysis

In order to analyze the time complexity of the algorithm, DNA one-time-pad encryption algorithms similar to our algorithm were selected to compare the total encryption time. The total encryption time consists of algorithm initialization, parameters encryption, self-assembly structure generation and data encryption time. It is assumed that the key generation time of the algorithm is *T*_1_, the encryption time for each set of parameters is *T*_2_, the generation time of each self-assembled structure is *T*_3_, and the time of each bit plaintext to generate ciphertext is *T*_4_. If the bit numbers of plaintext are *n*, the total encryption time comparison of the algorithms is shown in Table 7.

**Table 7:** Total encryption time comparison of the algorithms.

Literatures	Algorithm initialization time	Parameters encryption time	Self-assembly structure generation time	Data encryption time	Total encryption time
Ref. [26]			*nT*_3_	*nT*_4_	*n*(*T*_3_ + *T*_4_)
Ref. [29]			*nT*_3_	*nT*_4_	*n*(*T*_3_ + *T*_4_)
Ref. [30]	*T*_1_	*nT*_2_	*nT*_3_	*nT*_4_	*T*_1_ + *n*(*T*_2_ + *T*_3_+ *T*_4_)
Our algorithm	3*T*_1_	*T*_2_		*nT*_4_	3*T*_1_ + *T*_2_ + *nT*_4_

According to the four components of encryption time in Table 7, references [26] and [29] do not include algorithm initialization and parameter encryption time, and our algorithm does not include self-assembly structure generation time.

The algorithm initialization time in reference [30] is the key generation time *T*_1_. The initialization time of our algorithm is 3 times that of the algorithm in reference [30], which includes not only the key generation time, but also the selection time of the encoding mapping parameters and the generation time of the confusion encoding table. In terms of parameters encryption time, our algorithm is a fixed value *T*_2_. However, parameters encryption time in reference [30] is related to the bit numbers *n* of plaintext, which is *nT*_2_. References [26], [29] and [30] use self-assembled structures to construct logical operations. The generation time of each self-assembled structure *T*_3_ is uncontrollable and uncertain due to the influence of artificial operation and experimental conditions, while our algorithm does not include this process. And the generation time of self-assembled structure in the algorithms of these three literatures is proportional to the *n*. In addition, the data encryption time *nT*_4_ of each algorithm is also proportional to the bit numbers *n* of plaintext.

Although the initialization time of our algorithm is three times key generation time, it does not include the uncertain self-assembling structure generation time which is proportional to *n*. By comparing the total encryption time of these DNA one-time-pad encryption algorithms, our algorithm has relatively less encryption time and provides a reasonable time complexity.

### Algorithm performance features analysis

Ubaidurrahman *et al.* [35] proposed six efficient DNA cryptography properties in 2015, while Peng *et al.* [30] defined five performance parameters in 2018. In order to qualitatively compare the performance of algorithms, this paper defines the efficient performance features of DNA-based one-time-pad cryptography algorithms, as shown in Table 8. In addition, the performance features comparison of DNA-based one-time-pad algorithms are shown in Table 9.

**Table 8:** The features of effective DNA cryptography schemes.

Performance features	Definition
Complete character set encoding	The complete character set encoding requires that all plaintext elements including letters (uppercase, lowercase), numbers, and special characters can be converted into DNA sequence character sets.
Unique sequence for character encoding	The encoding of plaintext into DNA sequence is unique for every element of the character set in every session.
Dynamic encoding process	Different DNA sequence character sets can be randomly encoded for plaintext elements for each interaction session between sender and receiver.
Dynamic encryption process	The encryption and decryption algorithm should provide highly random encryption process, and the operations should include several random steps to generate different ciphertext for plaintext.
Biological encryption process	DNA encryption and decryption algorithm provide the physical isolation security of biological encryption by using DNA biotechnology.
Biological process simulation	DNA encryption and decryption algorithms should be based on biological processes or experimental techniques simulation to adapt to the digital computing environment.

**Table 9:** The features comparison of DNA-based one-time-pad schemes.

Literatures	Complete character set encoding	Unique sequence for character encoding	Dynamic encoding process	Dynamic encryption process	Biological encryption process	Biological process simulation
Ref. [13]	×	×	×	×	√	×
Ref. [24]	×	×	×	×	√	×
Ref. [26]	×	×	×	×	√	×
Ref. [27]	×	×	×	×	√	×
Ref. [28]	×	×	×	×	√	×
Ref. [25]	×	√	×	×	√	√
Ref. [29]	×	×	×	×	√	√
Ref. [23]	√	√	×	×	×	√
Ref. [31]	√	√	×	×	√	√
Ref. [14]	√	√	√	*	×	√
Ref. [30]	√	√	√	*	√	√
Our algorithm	√	√	√	√	√	√

^×^—Indication of minimum level of support.

^√^—Indication of acceptable level of support.

*—Partial fulfillment.

This article adopts the ASCII code combined with encoding mapping parameters *key*_*i*_, *i* = 0, 1, 2, satisfy the transition of plaintext elements to the binary random numbers and the transition of binary random numbers to base, to ensure the integrity and uniqueness of the encoding. In addition, the randomness of encoding mapping parameters *key*_*i*_, *i* = 0, 1, 2 and confusion encoding table parameter *Pk* ensures that every session can randomly encoding different DNA ciphertext and amino acid ciphertext for plaintext and parameters, thus realizing the dynamic encoding process. In this paper, the dynamic encryption process is provided by random selectivity of security and confounding parameters. In addition to the encoding randomness provided by encoding mapping parameters *key*_*i*_, *i* = 0, 1, 2 and confusion coding table parameter *Pk*, it also includes the randomness of Logistic map parameters *μ*_0_, *g*_0_, *μ*_1_, *g*_1_ and reassembly parameter *r*. After cutting and reassembling the DNA ciphertext sequence, the biosecurity is achieved through the synthetic DNA sequence and sequencing biotechnologies. In this scheme, the process of parameter encryption and decryption simulates the flow of genetic information from DNA to protein, which satisfies the biological process simulation and can resist the attack of mathematical analysis that rely on mathematical basis and cryptographic characteristics.

## Security analysis

### Key space analysis

A secure and effective one-time-pad algorithm should have a key space large enough to effectively resist brute force attacks. Most of the existing DNA-based one-time-pad algorithms rely on the biological experiment process, but the encoding is too simple. Once the experimental process is known, the algorithm is easy to be cracked. This paper proposes a cryptography scheme based on security parameters and sequencing technology. The encryption process depends on the selection of security parameters, and the security parameters are confused. In addition, high-throughput sequencing technology provides biosafety. In fact, the algorithm can provide high computational security under the condition that the key is leak. Assuming that the computational accuracy of the computer is 10^−16^, the actual key in the algorithm of this scheme includes parameters and their corresponding key spaces as follows:

1. Encoding mapping parameters *key*_*i*_, (*i* = 0, 1, 2).
  $$\begin{array}{c} S_{key}=S_{key_{0}}\times S_{key_{1}}\times S_{key_{2}}=24^{3}. \end{array}$$ (9)
2. Logistic map initial parameters *μ*_0_, *g*_0_, *μ*_1_, *g*_1_.
  $$\begin{array}{c} S_{{\text{g}}_{\text{0}}}=S_{{\text{g}}_{1}}\approx 10^{16},S_{\mu_{0}}=S_{\mu_{1}}\approx 0.5\times 10^{16}. \end{array}$$ (10)
3. Confusion encoding table parameter *Pk*.
  $$\begin{array}{c} S_{Pk}=256^{12}. \end{array}$$ (11)
4. DNA sequencing structure reassembly parameter *r*.
  $$\begin{array}{c} S_{r}=4^{40}. \end{array}$$ (12)

In summary, the total key space of algorithm is:

$$\begin{array}{c} \begin{array}{cc} & S=S_{key}\times S_{\mu_{0}}\times S_{\mu_{1}}\times S_{g_{0}}\times S_{g_{1}}\times S_{Pk}\times S_{r}\\ & \approx 24^{3}\times {\left(0.5\times 10^{16}\right)}^{2}\times {\left(10^{16}\right)}^{2}\times 256^{12}\times 4^{40}\\ & \approx 3^{3}\times 5^{64}\times 2^{247}. \end{array} \end{array}$$ (13)

Therefore, the key space of the algorithm is sufficient to effectively resist exhaustive attack. We compared the key space with some DNA image encryption algorithms that rely on mathematical security, as shown in Table 10. Our algorithm provides the same or even larger key space as the DNA image encryption algorithm without considering the biosafety.

**Table 10:** The key space comparison of DNA encryption algorithms.

Algorithms	Key space
Ref. [21]	1.92 × 10^126^
Ref. [22]	8.39 × 10^54^(*L* = 2)
Our algorithm	3^3^ × 5^64^ × 2^247^

### Sensitivity analysis

**Fig 13:**
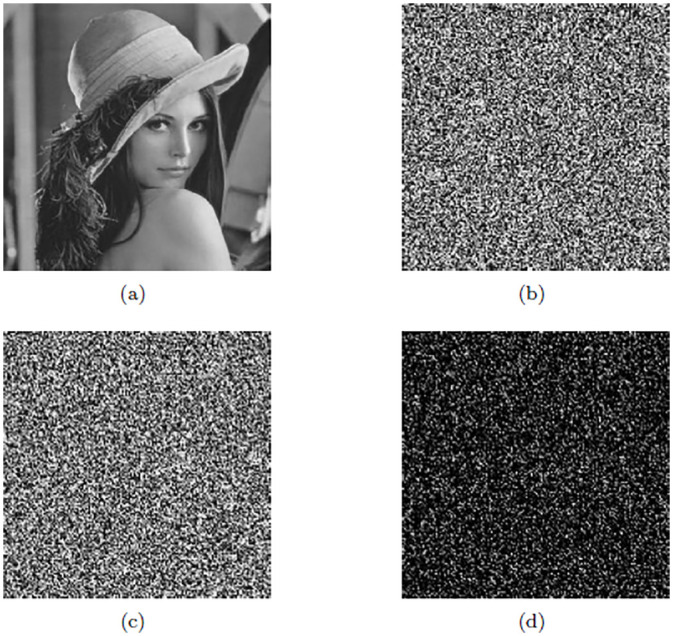
Key sensitivity of (*μ*_0_, *g*_0_). (a) Original Lena image. (b) Ciphered Lena image using (*μ*_0_, *g*_0_) = (3.78, 0.51). (c) Ciphered Lena image using (*μ*_0_, *g*_0_) = (3.780000000001, 0.510000000001). (d) Difference between (b) and (c).

In order to evaluate the key sensitivity of the proposed data encryption algorithm, the key sensitivity analysis method in literature [36] was referenced. We make one set of Logistic map initial parameters with a slightly change and the other parameters unchanged. The Lena gray image was selected as the source data and the original image is converted into ciphertext image with one-time-pad key *K* and Arnold map encryption. The original image in (a) of the Fig 13 was encrypted by (*μ*_0_, *g*_0_) = (3.78, 0.51) and (*μ*_0_, *g*_0_) = (3.780000000001, 0.510000000001), and the two ciphertext images obtained are shown in (b) and (c) of Fig 13 respectively. The difference of two ciphered images is 71.6% in (d) of Fig 13, which indicates the relatively high sensitivity of the (*μ*_0_, *g*_0_).

**Fig 14:**
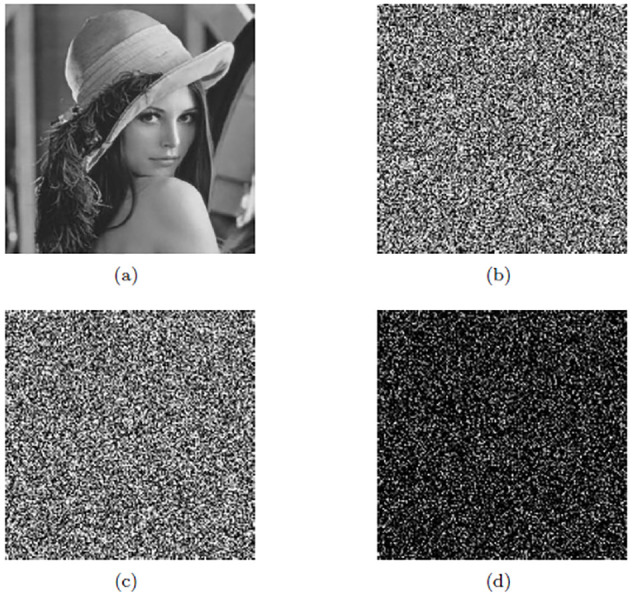
Key sensitivity of (*μ*_1_, *g*_1_). (a) Original Lena image. (b) Ciphered Lena image using (*μ*_1_, *g*_1_) = (3.92, 0.44). (c) Ciphered Lena image using (*μ*_1_, *g*_1_) = (3.920000000001, 0.440000000001). (d) Difference between (b) and (c).

For testing sensitivity of another set of Logistic map initial parameters. The Lena plaintext image in (a) of Fig 14 was encrypted by (*μ*_1_, *g*_1_) = (3.92, 0.44) and (*μ*_1_, *g*_1_) = (3.920000000001, 0.440000000001), and the two ciphertext images obtained are shown in (b) and (c) of Fig 14 respectively. The difference of two ciphered images is 72.3% in (d) of Fig 14, which indicates the relatively high sensitivity of the (*μ*_1_, *g*_1_).

We employ the NPCR (number of pixels change rate) and UACI (unified average changing intensity) which is defined in Eqs (14) and (15). The values of the two standards reflect the sensitivity of the encryption algorithm to plaintext image changes. The larger the values of NPCR and UACI, the more effective for the cryptosystem to resist differential attack.

$$\begin{array}{c} D\left(i,j\right)=\{\begin{array}{c} 1,c_{1}\left(i,j\right)\neq c_{2}\left(i,j\right)\\ 0,otherwise \end{array},NPCR=\frac{\sum_{ij}D(i,j)}{M\times N}\times 100\%. \end{array}$$ (14)

$$\begin{array}{c} UACI=\frac{1}{M\times N}[\sum_{ij}\frac{c_{1}\left(i,j\right)-c_{2}\left(i,j\right)}{255}]\times 100\%. \end{array}$$ (15)

The value of pixel (1,1) of the Lena image was changed to 0. Three sets of different Logistic initial parameters were used for encryption, and the obtained NPCR and UACI values are shown in Table 11.

**Table 11:** NPCR and UACR performance of ciphered Lena image.

(*μ*_0_, *g*_0_), (*μ*_1_, *g*_1_)	NPCR	UACI
(3.78,0.51),(3.92,0.44)	90.15	0.0138
(3.71,0.15),(3.82,0.43)	90.13	0.0106
(3,68,0.405),(3.92,0.44)	90.14	0.0167

Our NPCR and UACR values are not as well as the image encryption algorithms [21, 22]. However, the proposed data encryption algorithm includes key *K*, Arnold map, encoding mapping parameter *K*_0_ and reassembly parameter *r* encryption, we ensure the confidentiality of the data through multiple steps. In addition, we further improve the data security through high-throughput sequencing technology and parameter encryption algorithm. The simulation experiment is the test of the key and Arnold map encryption, and is only a part of the data encryption algorithm.

### Resistance attack analysis

The attack methods of cryptanalysts mainly include exhaustive attack method, statistical analysis method and mathematical analysis attack method. Firstly, the resistance attacks are analyzed according to the decoding method.

#### Resists exhaustive attack

The attacker uses the key in the key space to one by one demystify the obtained ciphertext. In this algorithm, each step of encryption and decryption is determined by security parameters, including Logistic map parameters *μ*_0_, *g*_0_, *μ*_1_, *g*_1_, encoding mapping parameter *key*_0_, and reassembly parameter *r*. Among them, the key is generated by Logistic map, the key value depends on two sets of initial parameters *μ*_0_, *g*_0_, *μ*_1_, *g*_1_, and the key space provided is 0.25 × 10^64^ when the calculation precision is 10^−16^. The selection probability of *key*_0_ is 1/24. The length of reassembly region added to each DNA ciphertext sequence was 40*nt*, and 4^40^ options were provided. In addition, the algorithm encrypts security parameters by confounding parameters, including 24^2^ choices provided by encoding mapping *key*_1_, *key*_2_ and the key space 256^12^ provided by confusion coding table parameter. Therefore, it is not feasible to decipher by exhaustive attack.

#### Resists statistical analysis attack

Attackers can break ciphertext by analyzing the statistical regularity of plaintext, ciphertext and key. In this algorithm, the random selection of security parameters and confounding parameters ensures that the same plaintext data generates different DNA ciphertext sequences and amino acid ciphertext sequences in every session, and the dynamic encryption process of the algorithm ensures that the attacker cannot obtain statistical laws from different sessions. The security of plaintext data depends on the choice of security parameters *μ*_0_, *g*_0_, *μ*_1_, *g*_1_, *key*_0_ and *r*, and Logistic map parameters *μ*_0_, *g*_0_, *μ*_1_, *g*_1_ generate the key. In the key sensitivity analysis of this paper, it can be seen that the key randomness of this algorithm is very well. Furthermore, the safety of security parameters depends on the dynamic generation of the confusion encoding table, the selection of *key*_1_, *key*_2_ and confusion encoding table parameter *Pk*. In the frequency analysis of parameters, there is no statistical similarity between parameters plaintext and ciphertext.

#### Resists mathematical analysis attack

Attackers can break the ciphertext by solving it mathematically. The data encryption algorithm in this algorithm includes not only the XOR operation from binary plaintext to binary ciphertext, but also the confounding operation of binary ciphertext to DNA ciphertext and DNA ciphertext to DNA synthesis sequence. The whole process is random and does not rely solely on mathematical calculation. Moreover, the parameter encryption algorithm simulates the process of biological genetic information flowing from DNA to protein without relying on mathematical difficulties and cryptographic characteristics. Multistep confounding operations make the cryptographic algorithm more random. Therefore, even if the attacker obtains the DNA ciphertext sequence, the security parameters cannot be decrypted by mathematical analysis. Moreover, plaintext data cannot be decrypted without security parameters.

Assuming that the attacker knows all encryption algorithms, the attack types of encryption system can be divided into four types according to the attacker’s mastery of data resources such as plaintext and ciphertext. In addition to the analysis of chosen plaintext attack in literature [37], we also make a detailed analysis of the other three types of attacks.

#### Resists only ciphertext attack

The attacker can only analyze the intercepted ciphertext to obtain plaintext or key. In this algorithm, the publicly transmitted ciphertext consists of amino acid parameters ciphertext and synthetic DNA mixtures. Parameters ciphertext is generated using confounding parameters encryption, where the selection probability provided by the encoding rules *key*_1_, *key*_2_ is 1/24^2^, the selection probability provided by the dynamic generation of confusion encoding table is $1/A_{20}^{16}\times A_{20}^{16}$, and the selection probability provided by the confusion encoding table parameter *Pk* is 1/256^12^. Therefore, the probability of cracking the amino acid ciphertext without considering the encryption algorithm is very small. It would also be nearly impossible for an attacker to decipher DNA sequences. First, the attacker needs to know the adapter information of the DNA sequence. In addition, random primer sequencing without knowing the adapter would not only be costly, but multiple amplification reactions would contaminate the DNA mixtures.

#### Resists known plaintext attack

The attacker intercepts some pairs of plaintexts and ciphertexts to break the key or algorithm. In the case that the attacker obtains some pairs of plaintext data and corresponding DNA ciphertext sequences. Because data encryption takes different security parameters in every session, each step of the plaintext data generation DNA ciphertext sequence provides security. Therefore, it is unrealistic to crack encryption algorithms and keys based on some corresponding plaintext data and DNA ciphertext sequences. It is assumed that the attacker has obtained some pairs of security parameter plaintexts and corresponding amino acid ciphertexts. Since each security parameter encryption use different encoding mapping parameters *key*_1_, *key*_2_ and confusion encoding tables, it not only has one-time-pad characteristics, but also simulates the biological operation process to make the algorithm more random. Therefore, it is relatively safe for known plaintext attacks.

#### Resists chosen plaintext attack

Besides getting some corresponding ciphertext, the attacker has analyzed and obtained more information related to the key. According to the comparison of DNA ciphertext sequence and plaintext data, the operation processes of data encryption and decryption algorithm are obtained. It is unable to get encoding mapping parameter *key*_0_ and the key through security parameters because its success is without cracking security parameters. It is difficult to decipher the DNA ciphertext sequence during the next decryption.

#### Resists chosen ciphertext attack

The attacker can select some ciphertext and get the corresponding plaintext. It is assumed that the attacker has mastered the data encryption algorithm, the selected DNA ciphertext sequence, the decrypted plaintext, and the key of the selected part. This one-time-pad algorithm uses different Logistic map parameters to generate the key for each encryption process. Even if the key is cracked this time, it cannot be used for the next decryption. Besides the key, the mapping parameter *key*_0_ are also different in every session and cannot be used in the next decryption. In addition, DNA ciphertext sequences were segmented and primers were added at both ends. If you don’t know the index order, you can’t splice the information correctly, which is a big obstacle to decipher the DNA ciphertext.

### Biological security analysis

**Fig 15:**
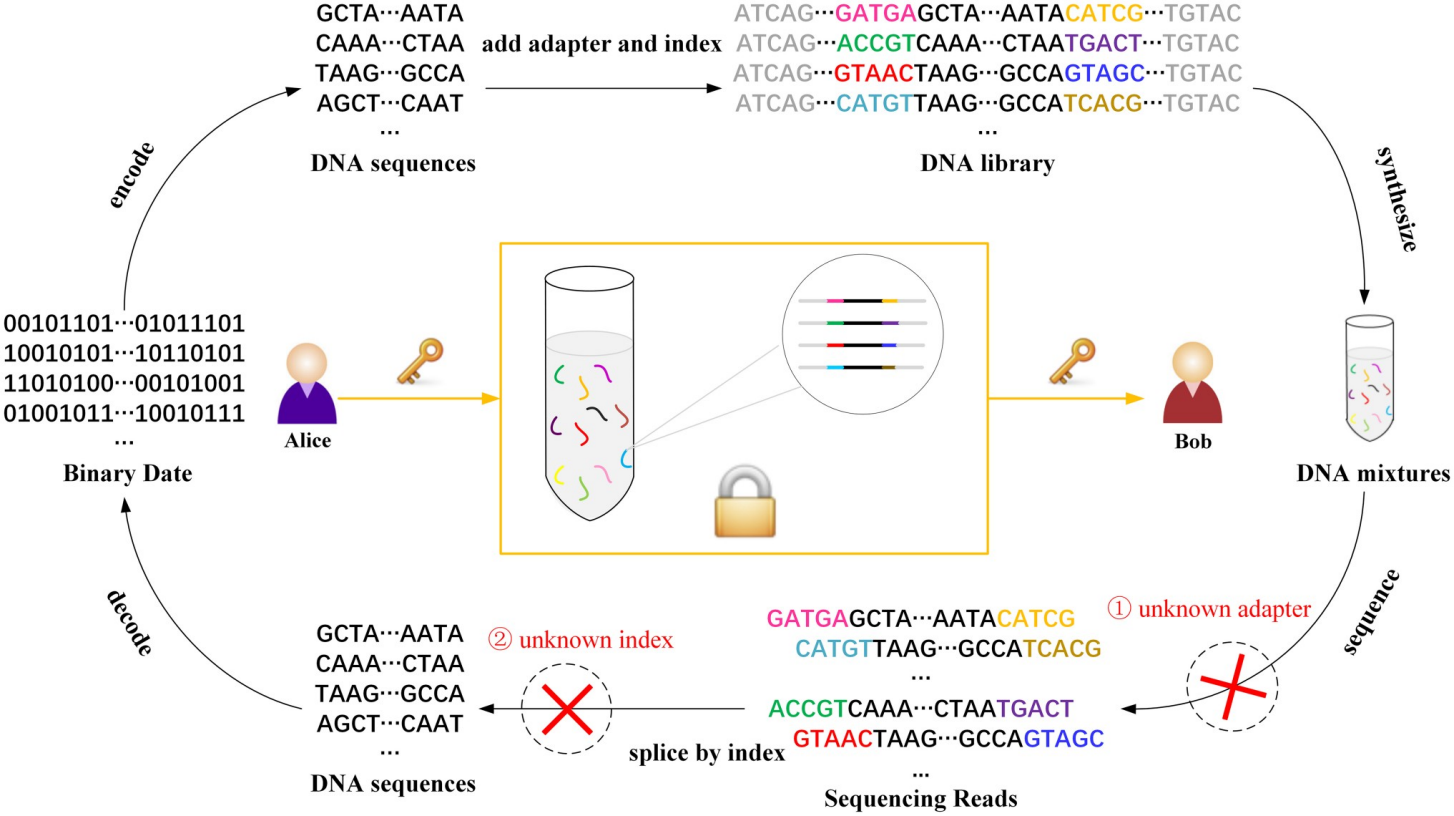
The biological encryption process for the data.

High-throughput sequencing technology has experienced more than ten years of rapid development. At present, the second-generation sequencing equipment has greatly improved in flux and accuracy. Meanwhile, the sequencing cost has also been greatly reduced, making it the mainstream of commercial sequencing. This is a great opportunity for the development of DNA storage and DNA cryptography. In the existing DNA-based one-time-pad algorithms design, some encryption algorithms are purely based on biological experiments, and the operation process is complex and uncontrollable, which is not suitable for the computing environment of digital application. Some encryption algorithms only simulate DNA biological characteristics, but do not provide biological security. In this algorithm, the DNA ciphertext sequence obtained by data encryption is segmented with random adapter and index. After artificial synthesis, it is publicly transmitted. During decryption, DNA ciphertext sequence was obtained by high-throughput sequencing and index splicing. The biological encryption processes for the data are shown in Fig 15.

This scheme combines DNA frontier technology with cryptographic algorithm, which not only guarantees computational security but also provides controllable biological security:

In case ➀, even if the attacker obtains the entire DNA mixtures, sequence information cannot be obtained by sequencing without knowing the adapter sequence. In addition, when the attacker uses random primers for PCR sequencing regardless of cost, multi-sequencing amplification can contaminate the DNA mixtures.

In case ➁, even if the sequencing is successful, the correct order of DNA sequences cannot be obtained because the index sequence is unknown.

## Conclusion

The one-time-pad cipher algorithm based on confusion mapping and DNA technology is divided into two parts: data and parameter encryption algorithm. Each step in the process of data encryption is determined by security parameters, among which the key generation parameters not only realize one-time-pad encryption characteristics but also facilitate key management, encoding mapping parameters ensure the randomness of binary sequence and DNA sequence transformation, and the addition of reassembly parameter is the premise of DNA sequence segmentation and splicing. In addition, the combination of synthetic and high-throughput DNA sequencing technologies with cryptographic algorithms greatly improves the security of the scheme. In the process of parameter encryption, amino acid ciphertext is generated according to confounding parameters, which simulates the flow of biological genetic information from DNA to protein, increasing the randomness of the algorithm. In this paper, the parameters preparation, plaintext encryption and decryption process, and ciphertext transmission in the algorithm are described in detail. By analyzing the performance and security of the algorithm, it can be seen that the algorithm in this paper not only provides computational security but also biological security. The security of cipher algorithm has been greatly improved and can resist various attacks.
